# Vaginal *Acinetobacter* infection as a cause of cervical collagenolysis

**DOI:** 10.3389/fcimb.2026.1845575

**Published:** 2026-06-19

**Authors:** Qiuyu Sun, Yan Cai

**Affiliations:** Department of Obstetrics and Gynecology, The Fourth Affiliated Hospital of Harbin Medical University, Harbin, Heilongjiang, China

**Keywords:** *Acinetobacter*, collagen fiber dissolution, MMP-1, Snai2, vaginal flora imbalance

## Abstract

**Background:**

The vaginal microbiota demonstrates considerable heterogeneity and is shaped by factors such as race, geographic location, and individual variation. Although its central role in maintaining host health is well established, the precise mechanisms through which it contributes to the onset and progression of preterm birth remain incompletely understood and continue to be debated.

**Objective:**

This study aimed to characterize the vaginal microbiota of pregnant women who experienced preterm birth compared with those with full-term pregnancies. Through both *in vivo* and *in vitro* experiments, the study investigated whether vaginal pathogenic bacteria associated with preterm birth can trigger cervical collagen fiber degradation and induce the expression of MMP-1.

**Method:**

High-throughput sequencing was used to analyze the vaginal microbial communities of women with preterm and full-term pregnancies. On gestational days (GD) 14 and 15, pregnant C57BL/6 mice received intravaginal inoculations of 50 μL containing either 5 × 10^8^ CFU/mL *Acinetobacter*, 5 × 10^8^ CFU/mL *Lactobacillus gasseri*, or sterile phosphate-buffered saline (PBS). Cervical tissues were collected on GD16. Collagen fiber degradation was evaluated using Sirius red staining, and MMP-1 expression was assessed by immunohistochemistry. Hcer Epic cells were co-cultured with *Acinetobacter* or *Lactobacillus gasseri*, after which MMP-1 expression was measured using ELISA, qPCR, Western blotting, and immunofluorescence staining. The transcriptional regulatory role of Snai2 in controlling MMP-1 expression was examined using chromatin immunoprecipitation (ChIP), dual-luciferase reporter assays, and Western blot analysis.

**Results:**

*Acinetobacter* was identified as a characteristic bacterium in the vaginal microbiota of women with preterm birth. Experimental results showed that this bacterium induced cervical collagen fiber degradation in pregnant mice and increased MMP-1 expression in both the mouse model and Hcer Epic cells. Mechanistic analyses revealed that Snai2 binds to the GCACCTGT motif in the MMP-1 promoter. Furthermore, *Acinetobacter* might enhance MMP-1 expression through the activation of Snai2. These findings are limited to the specific strains examined and warrant further investigation across additional isolates.

**Conclusions:**

These findings indicate that *Acinetobacter* promotes cervical collagen fiber degradation and elevates MMP-1 expression in both animal and cellular models, suggesting that this bacterium may contribute to cervical collagen remodeling and potentially play a pathogenic role in the development of preterm birth.

## Introduction

Preterm birth is a major contributor to mortality around the time of birth and during early childhood worldwide (GBD 2019 Under-5 Mortality Collaborators, 2021). According to data reported in 2023, the global preterm birth rate is approximately 9.9% (Ohuma et al., 2023). Complications associated with premature birth not only severely compromise child health but also place a substantial economic burden on families and healthcare systems. Despite extensive international research into the pathogenesis of preterm birth, effective strategies for its prevention and treatment remain limited. Recent studies suggest that pregnant women with an imbalanced vaginal microbiota have a higher risk of preterm birth (Fettweis et al., 2019; Bayar et al., 2020; Gudnadottir et al., 2022; Li et al., 2024; Schuster et al., 2024). However, other investigations have reported no significant reduction in preterm birth rates following therapeutic interventions targeting vaginal microbiota (Reid and Bocking, 2003; Abramovici et al., 2015). These inconsistent findings indicate that the relationship between the vaginal microbiota and preterm birth requires further clarification.

The vaginal microbiota represents an important component of the human microbiome and, under healthy conditions, is typically dominated by *Lactobacillus* species. These bacteria help prevent colonization by pathogenic microorganisms and protect against viral and bacterial invasion by producing lactic acid, hydrogen peroxide (H^2^O^2^), bacteriocins, and other antimicrobial substances (Saraf et al., 2021). Compared with non-pregnant women, pregnant women generally show reduced diversity but increased stability in their vaginal microbiota, which helps maintain a healthy pregnancy environment (Freitas et al., 2017). Previous studies have indicated that a decrease in the relative abundance of *Lactobacillus* species, accompanied by an increase in opportunistic pathogens during pregnancy, may elevate the risk of adverse outcomes such as gestational vaginitis, preterm birth, and premature rupture of membranes (Petricevic et al., 2014; Dunlop et al., 2015; Zhao et al., 2021; Li et al., 2024). However, significant racial and regional variations in vaginal microbiota composition often yield inconsistent findings across studies. Moreover, research on individual bacterial species has largely focused on well-known pathogens such as *Gardnerella vaginalis*. In comparison, systematic investigations of differentially expressed bacterial species identified through high-throughput sequencing remain relatively limited and warrant further exploration.

The cervix, which forms the anatomical passage between the uterus and the vagina, functions both as a barrier separating the uterine cavity from the external environment and as a dynamic structure that plays a critical role throughout pregnancy. Cervical collagen fibers are the principal determinant of cervical rigidity. During pregnancy, high concentrations of collagen fibers form a dense reticular framework that mechanically supports the fetus and associated gestational tissues. In the weeks preceding delivery, the cervix gradually undergoes a process known as cervical remodeling, during which its stiffness decreases while sufficient tensile strength is maintained to allow normal parturition (Myers et al., 2015; Nallasamy and Mahendroo, 2017). The degradation and remodeling of cervical collagen fibers are primarily regulated by matrix metalloproteinases (MMPs). At the same time, hormonal signals and inflammatory mediators influence this process by activating MMPs and inhibiting tissue inhibitors of metalloproteinases (TIMPs) (Timmons et al., 2010). As a result, abnormal activation of MMPs during pregnancy may lead to premature cervical remodeling, increasing the risk of late miscarriage and preterm birth.

In this study, high-throughput sequencing was used to characterize the vaginal microbiota of women who experienced preterm birth and to identify bacterial species that differ from those in normal pregnancies. Furthermore, we investigated whether these differential bacteria could promote cervical collagen degradation and explored the potential regulatory mechanisms underlying this process.

## Materials and methods

### Clinical samples

At the initial visit, vaginal secretions from the posterior fornix were collected under strict aseptic conditions from 20 pregnant women diagnosed with preterm birth or threatened preterm birth between 24 and 27^+6^ weeks of gestation. Immediately after collection, each sample was suspended in phosphate-buffered saline and stored at −80 °C until further analysis. Threatened preterm birth was diagnosed based on the presence of regular uterine contractions (≥4 within 20 minutes or ≥8 within 60 minutes) together with a cervical length ≤20 mm measured by transvaginal ultrasound, in the absence of cervical dilation (Early prenatal care and prevention guidelines (2024 edition)). Written informed consent was obtained from all participants before sample collection, and the study protocol was approved by the Ethics Committee of the Fourth Affiliated Hospital of Harbin Medical University (Approval No. 2024-DWSYLLCS-38).

### Murine vaginal infection model

Six to eight-week-old C57BL/6 mice were used for mating at a ratio of two females to one male. Pregnancy was confirmed by the detection of a vaginal plug, and the day of plug observation was designated as gestational day 0 (GD0). Pregnant females were then randomly assigned to three groups. On GD14 and GD15, mice were anesthetized and intravaginally inoculated with 50 μL of either (1) *Acinetobacter* sp. suspension (5 × 10^8^ CFU/mL), (2) *Lactobacillus gasseri* suspension (5 × 10^8^ CFU/mL), or (3) sterile phosphate-buffered saline (PBS). Cervical tissues were collected on GD16 for subsequent analyses (Gilbert et al., 2013; Sierra et al., 2018; Joseph et al., 2023).

### Cell lines

Human normal cervical epithelial cells (Hcer Epic) were obtained from Otwo Biotech (Shenzhen, China). The cells were cultured in DMEM supplemented with 10% fetal bovine serum (FBS) and 1% penicillin–streptomycin and maintained under standard conditions at 37 °C in a humidified incubator with 5% CO^2^.

### Bacterial cultivation

The bacterial strains used in this study, *Acinetobacter* sp. (BNCC 337486) and *Lactobacillus gasseri* (BNCC 135322), were obtained from the Engineering Technology Research Center of Industrial Microbial Strains of Henan Province (BNCC, China). *Acinetobacter* sp. was cultured aerobically at 37 °C for 18–24 h in Nutrient Broth medium (BNCC, China), whereas *Lactobacillus gasseri* was cultivated under microaerobic conditions at 37 °C for 24–48 h in de Man, Rogosa and Sharpe (MRS) medium (BNCC, China). All experimental results were based on the tested strains.

### DNA extraction and control

The integrity of the extracted DNA was first evaluated by 1% agarose gel electrophoresis. DNA purity was subsequently assessed using a Nano Drop 200 spectrophotometer (Thermo Fisher, USA), with acceptable OD260/280 ratios ranging from 1.7 to 1.9, and an initial concentration measurement was obtained. Accurate quantification was then performed using a Qubit^®^ 3.0 Fluorometer (Life Technologies, USA).

### 16S rRNA high-throughput sequencing

The V3–V4 hypervariable regions of the bacterial 16S rRNA gene were amplified using the universal primers 341F (5′-ACTCCTACGGGAGGCAGCAG-3′) and 806R (5′-GGACTACHVGGGTWTCTAAT-3′). PCR amplification was carried out in a 50 μL reaction mixture containing 2 μL of template DNA, 1 μL each of forward (P5) and reverse (P7) primers, 10 μL of 5× PrimeSTAR GXL Buffer, 1 μL of PrimeSTAR GXL DNA Polymerase, 4 μL of dNTP mixture, and 31 μL of nuclease-free water. The PCR program included an initial denaturation at 95 °C for 3 min, followed by 35 cycles of amplification (95 °C for 45 s, 47 °C for 45 s, and 72 °C for 45 s), with a final extension at 72 °C for 10 min. The resulting amplicons were purified using AMPure XP beads and eluted in Elution Buffer. After appropriate labeling, sequencing libraries were constructed. Library concentrations were measured using the NGS™ dsDNA HS Assay Kit on a Qubit^®^ 3.0 Fluorometer (Invitrogen, USA). Libraries that met the required quality standards were then sequenced on an Illumina NovaSeq platform using a 250-bp paired-end strategy.

### Bioinformatics and statistical analysis

Raw paired-end reads were processed using QIIME2 (v2023.5) with the DADA2 plugin. Quality filtering parameters were: truncation length 250 bp for both forward and reverse reads, max expected errors (max EE) 2.0, and truncation quality score (trunc_q) 2. Chimeras were removed using the consensus method. The resulting amplicon sequence variants (ASVs) were taxonomically classified using the feature-classifier classify-sklearn plugin against the SILVA 138 database (95% OTU similarity) with a confidence threshold of 0.7. Alpha diversity and beta diversity were computed within QIIME2. Differential abundance analysis was performed using DESeq2 (R v4.2.0), with FDR-adjusted *p* < 0.05 considered significant.

### Sirius Red staining

Mouse cervical tissues fixed in 4% paraformaldehyde for 24 h were embedded in paraffin and sectioned at a thickness of 6 μm. Collagen staining was then performed using a commercially available Sirius Red staining kit (60415es50, YeaSen, China) in accordance with the manufacturer’s instructions.

### Immunohistochemistry

Mouse cervical tissues fixed in 4% paraformaldehyde for 24 h were embedded in paraffin and sectioned at a thickness of 5 μm. Immunohistochemical staining was performed using an HRP-labeled polymer two-step detection system (EnVision™, Dako, USA; species specificity: anti-rabbit). The sections were incubated with a rabbit anti-mouse MMP-1 polyclonal antibody (Abmart, China, PA1747; dilution 1:200) in PBS containing 1% BSA overnight at 4 °C, and images were captured using a light microscope with a digital camera.

### Hydroxyproline assay

The hydroxyproline (HYP) content in mouse cervical tissues collected after modeling was determined using a commercially available assay kit (Sangon Biotech, China; Cat# D799574-0100) in accordance with the manufacturer’s instructions.

### Cell death assay

Hcer Epic cells were seeded in 96-well plates at a density of 5 × 10^4^ cells per well in DMEM supplemented with 10% heat-inactivated FBS, without antibiotics. The following day, the cells were co-cultured with *Lactobacillus gasseri* or *Acinetobacter* at concentrations ranging from 10^4^ to 10^8^ CFU per well for 24 h. Afterward, CCK-8 reagent (Biosynthesis Biotechnology, BA00208, Beijing) was added to each well at 10% of the total volume, and the plates were incubated at 37 °C with 5% CO^2^ for 1 h. Optical density was then measured at 450 nm using a microplate reader.

After co-culture with bacteria as described above, the culture medium was carefully removed, and the cell monolayer was gently washed twice with 200 μL of PBS to remove non-adherent bacteria and extracellular DNA/RNA.A working staining solution was prepared fresh by mixing 50 μL of ready-to-use PI staining solution (KeyGEN BioTECH, KGA1813-10) and 2 μL of SYTO staining solution (KeyGEN BioTECH, KGE2503-500) per well (for a 96-well plate). The mixture was added to each well, and the plate was incubated for 15 minutes at room temperature in the dark. After incubation, cells were immediately observed using a fluorescence microscope (PI: Ex/Em 535/615 nm; SYTO: Ex/Em 488/530 nm). Live cells appear green, while dead cells exhibit both green and red fluorescence.

### Enzyme-linked immunosorbent assay

The levels of MMP-1 in the culture supernatant following 24 h of co-culture with *Lactobacillus gasseri* or *Acinetobacter* sp. were measured using an ELISA kit (mlbio, China, ml038199) according to the manufacturer’s instructions.

### RNA isolation and qPCR

Total RNA was extracted from cells using the BioFlux Total RNA Extraction Kit (BioFlux, China, BSC52S1). The purity and concentration of RNA were determined by measuring the A260/A280 ratio with a NanoDrop™ spectrophotometer. Reverse transcription was then carried out to synthesize cDNA using the SEVEN First Strand cDNA Synthesis Kit (SEVEN, China, SM136). Quantitative PCR (qPCR) was subsequently performed on a QuantStudio™ 5 Real-Time PCR System with the SEVEN SYBR Green qPCR Master Mix (SEVEN, China, SM143). Relative gene expression levels were calculated using the 2^−ΔΔCt^ method, with GAPDH serving as the internal control. The following primers were used: human GAPDH (forward: 5’-CCACTCCTCCACCTTTGAC-3’, reverse: 5’-ACCCTGTTGCTGTAGCCA-3’); human MMP-1 (forward: 5’-ACCCTGAAGGTGATGAAGCA-3’, reverse: 5’-AGTGAGGACAAACTGAGCCA-3’);human Snai2(forward: 5’-CTCTCTCCTCTTTCCGGATACT-3’, reverse: 5’-GCTTGGACTGTAGTCTTTCCTC-3’).

### Immunofluorescence assay

After 24h of co-culture with *Lactobacillus gasseri* or *Acinetobacter* sp., Hcer Epic cells were processed for immunofluorescence staining. The cells were incubated with a rabbit anti-human MMP-1 polyclonal antibody (Abmart, China, PA1747; dilution 1:200) in PBS containing 1% BSA overnight at 4 °C. After washing, detection was performed using a FITC-conjugated goat anti-rabbit IgG secondary antibody (ZSGB-BIO, China, catalog number ZF0311; dilution 1:100) in PBS containing 1% BSA for 1h at room temperature in the dark. Cell nuclei were counterstained with DAPI (Solarbio, China, catalog number C0060; dilution 1:1000) for 5 min at room temperature in the dark. Images were captured using a fluorescence microscope with appropriate filter sets (FITC: Ex/Em 488/530 nm; DAPI: Ex/Em 358/461 nm).

### Western blotting analysis

Total protein lysates were prepared using RIPA buffer supplemented with a protease and phosphatase inhibitor cocktail. Equal amounts of protein (50 μg per lane) were separated by 10% SDS-PAGE and subsequently transferred onto nitrocellulose (NC) membranes. The membranes were then incubated overnight at 4 °C with primary antibodies against MMP-1 (Abmart, PA1747, 1:1000), Snai2 (Cell Signaling Technology, 9585, 1:1000), TNF-α (Proteintech, 26405-1-AP, 1:1000), and NF-κB p65 (Proteintech, 82335-1-RR, 1:1000). For normalization, the membranes were reprobed with antibodies against β-actin and GAPDH, which served as loading controls.

### Luciferase assay

The coding sequence of Snai2 and the 2000 bp upstream promoter region of MMP-1 were amplified and cloned into pCAG-IRES-GFP and pGL4.11[luc2P], respectively. The predicted Snai2 binding site in the MMP-1 promoter was mutated from GCACCTGT to GCAGGTGT using site-directed mutagenesis. Hcer Epic cells were co-transfected with the Snai2 overexpression plasmid, MMP-1 luciferase reporter plasmid, and pGL4.74[hRluc-TK] at a ratio of 30:15:1 using KeygenMax3000(KeyGEN BioTECH, China, KGA9705-1.5). Luciferase activity was measured 48h after transfection using the Dual-Luciferase Reporter Assay System (Promega, E1910).

### Chromatin immunoprecipitation assay

The ChIP assays were carried out using the BeyoChIP™ Kit with Protein A/G magnetic beads (Beyotime, China, P2080S) in accordance with the manufacturer’s instructions. Cross-linked chromatin was first sonicated to generate DNA fragments of approximately 200–500 bp. The fragmented chromatin was immunoprecipitated with an antibody against Snai2, and normal rabbit IgG served as the negative control. The precipitated DNA was subsequently purified and analyzed by qPCR. The following primers were used: (forward: 5’-GCCCAGGCTGATCTTGAACT-3’, reverse: 5’-TGTTCGGCACCTGTACTGAC-3’).

### Oligonucleotides transfections

Small interfering RNAs (siRNAs) targeting Snai2(sense: 5’-CAUUAGUGAUGAAGAGGAATT-3’), along with the corresponding control siRNA, and the Snai2 overexpression vectors with their control vectors, were synthesized by Kaiji Biotechnology Co. Ltd. (Jiangsu, China). The construct was verified by Sanger sequencing. Transfection of Hcer Epic cells were carried out using the KeygenMax3000 transfection reagent (KeyGEN BioTECH, China, KGA9705-1.5) according to the manufacturer’s instructions.

### Statistical analysis

All data were analyzed using GraphPad Prism 9.5 (La Jolla, CA, USA). Continuous variables with a normal distribution (assessed by Shapiro–Wilk test) are presented as the mean ± SD. Differences between two groups were evaluated using independent-samples t-tests. Comparisons among multiple groups were performed using one-way ANOVA, followed by Tukey’s honest significant difference (HSD) *post-hoc* test for multiple pairwise comparisons. Levene’s test was used to assess homogeneity of variances, and equal variances were assumed (*p*> 0.05). Categorical variables were analyzed using the chi-square test. A value of *p* < 0.05 was considered statistically significant.

## Results

### *Acinetobacter* is a vaginal microorganism marker for premature pregnant women

To characterize the vaginal microbiota in pregnant women with preterm birth, vaginal posterior fornix secretions were collected from 20 women with preterm birth and 20 women with uncomplicated pregnancies between 24 and 27^+6^ weeks of gestation. The collected samples were analyzed by 16S rRNA gene sequencing. All participants had resided in the sampling region for an extended period. The general demographic characteristics of the study population are summarized in **Table 1**.

**Table 1:** General condition of the subjects.

Characteristic	Control	Case	Z/χ²/t	*P*
**Age**	31.1 ± 2.99	30.8 ± 3.41	-0.206	0.837
**Height**	163.3 ± 5.28	164.53 ± 5.39	-0.726	0.473
**Weight**	60.5 ± 9.89	62.63 ± 7.85	-0.753	0.456
**Weeks**			6.473	0.091
24-24w6	3	8		
25-25w6	3	6		
26-26w6	7	3		
27-27w6	7	3		
**History of gestation**			4	0.549
G1P0	10	14		
G2P0	6	3		
G3P0	2	1		
G4P0	1	1		
G2P1	1	0		
G3P1	0	1		
**Education background**			5.276	0.509
primary school	0	1		
junior high school	1	3		
high school	1	2		
bachelor degree	13	7		
associate degree	3	4		
technical school	0	1		
graduate student	2	2		

*Bold values indicate statistical significance (*p* < 0.05).

**Figure 1:**
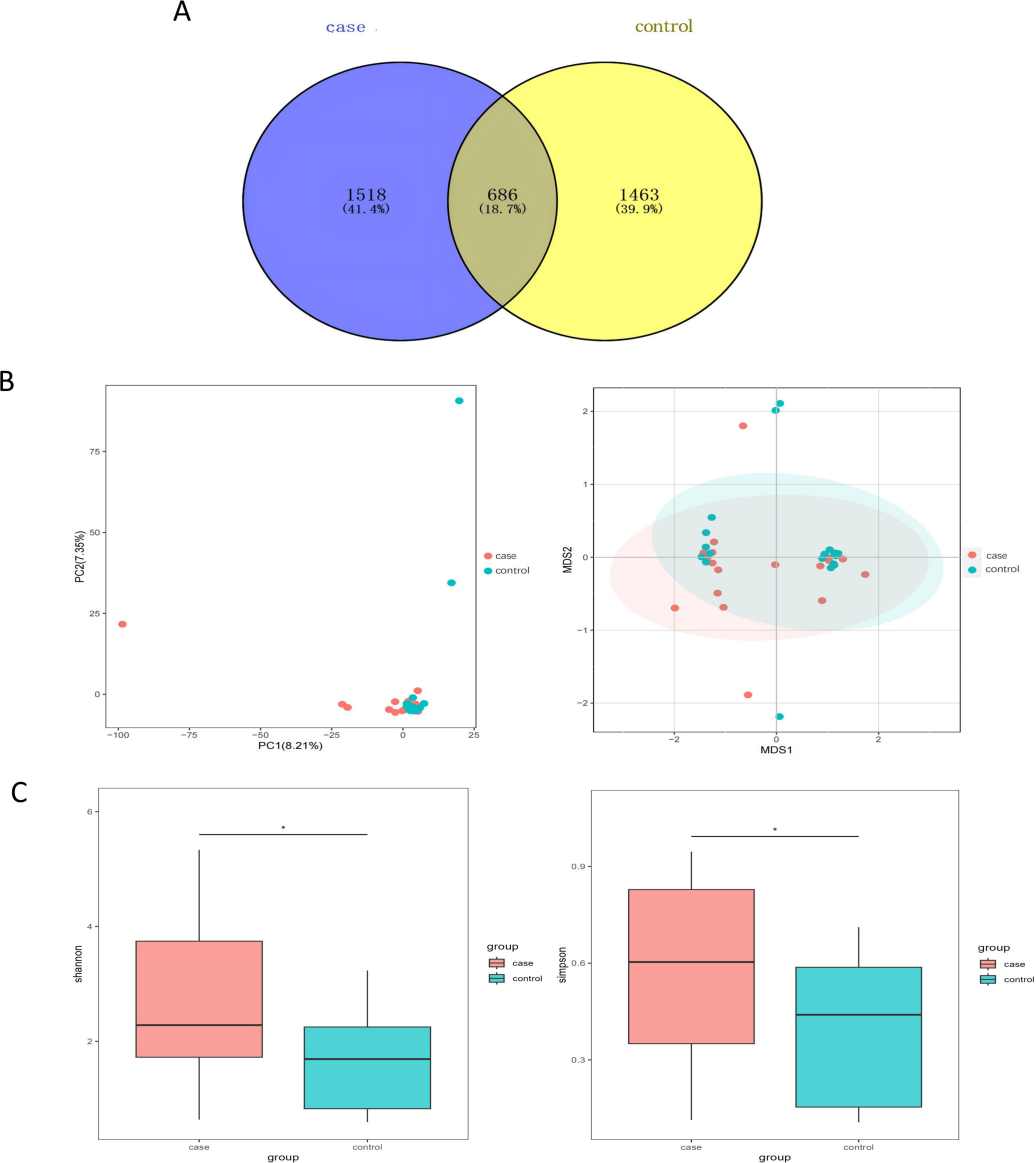
Diversity analysis of vaginal microbiota between the preterm and control groups based on 16S rRNA sequencing. Vaginal secretion samples were collected from normal pregnant women (control group, n=20) and women with preterm labor (preterm group, n=20). Sequencing was performed targeting the V3-V4 region of the 16S rRNA gene, and amplicon sequence variants (ASVs) were clustered at 99% sequence similarity **(A)**. β-diversity analysis based on BrayCurtis distance visualized by principal coordinate analysis (P CoA) and non-metric multidimensional scaling (NMDS). Each point represents an individual sample; red indicates the preterm group, blue indicates the control group. **(B)**. α-diversity was evaluated using the Simpson index and the Shannon index. Both the Simpson index and the Shannon index were significantly higher in the preterm group compared to the control group (*p* < 0.05) **(C)**.

The sequencing results identified 686 amplicon sequence variants (ASVs) shared by both groups, while 1,518 ASVs were unique to the preterm birth group and 1,463 ASVs were specific to the control group (**Figure 1A**). β-diversity was assessed using principal coordinate analysis (P CoA) and non-metric multidimensional scaling (NMDS) based on the Bray-Curtis distance. The two groups overlapped extensively without clear clustering separation, suggesting that the overall vaginal microbial community structures were similar between the preterm and control groups (**Figure 1B**). α-diversity was evaluated using the Simpson and Shannon indices. Compared with women with normal pregnancies, both the Simpson and Shannon diversity indices of the vaginal microbiota were significantly higher in the preterm birth group (*p* < 0.05; **Figure 1C**).

**Figure 2:**
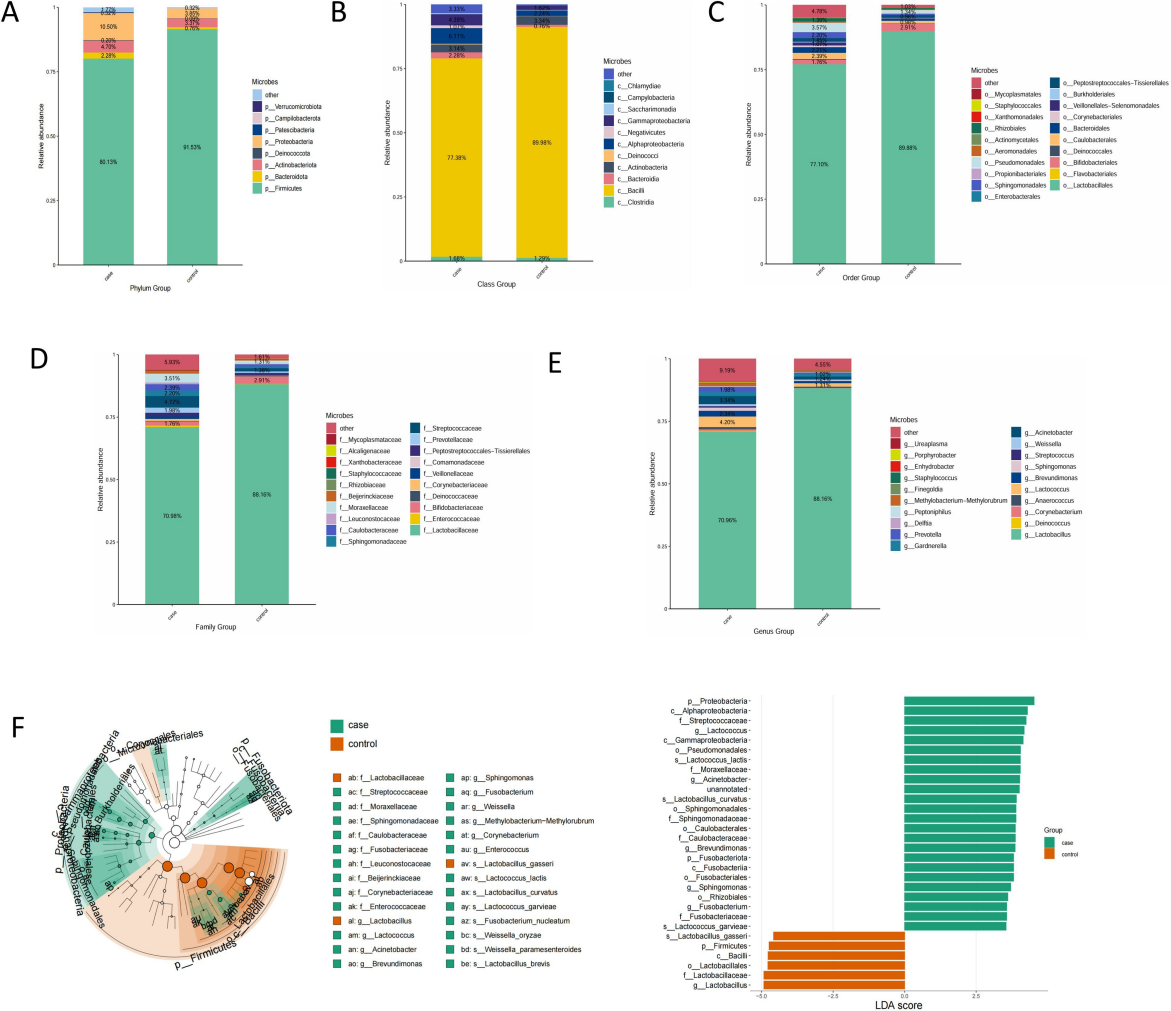
Relative species abundance at different taxonomic levels and Lef Se analysis between the preterm and control groups. Taxonomic analysis at the phylum **(A)**, class **(B)**, order **(C)**, family **(D)**, and genus **(E)** levels revealed a reduced relative abundance of *Lactobacillus* and an increased relative abundance of several opportunistic pathogens in the preterm birth group. Linear discriminant analysis (LDA) effect size (Lef Se) was performed to identify discriminatory microbial taxa (LDA score ≥ 4.0). The results identified *Lactobacillus gasseri* as the characteristic microorganism in the preterm group and *Acinetobacter* spp. as the characteristic microorganism in the control group. **(F)**.

Further taxonomic analysis at the phylum, class, order, family, and genus levels showed that the relative abundance of *Lactobacillus* was reduced in the preterm birth group compared with the control group (genus level: 70.96% vs. 88.16%). In comparison, several opportunistic or potentially pathogenic genera, including *Acinetobacter*, *Brevundimonas*, and *Prevotella*, demonstrated increased relative abundance (genus level: 3.34% vs. 1.24%; 2.34% vs. 0.96%; and 1.98% vs. 0.56%, respectively) (**Figures 2A–E**).

Linear discriminant analysis effect size (Lef Se) identified distinct microbial biomarkers distinguishing the two groups (*p* < 0.05, LDA score ≥ 4.0). In the normal pregnancy group, the key biomarker taxa at the phylum-to-species levels included *Firmicutes*, *Bacilli*, *Lactobacillales*, *Lactobacillaceae*, *Lactobacillus*, and *Lactobacillus gasseri*. In comparison, the preterm birth group was characterized by biomarker taxa from the phylum to genus levels, including *Proteobacteria*, *Gammaproteobacteria*, *Pseudomonadales*, *Moraxellaceae*, and *Acinetobacter* (**Figure 2F**).

### *Acinetobacter* induces the dissolution of cervical collagen tissue in mice

**Figure 3:**
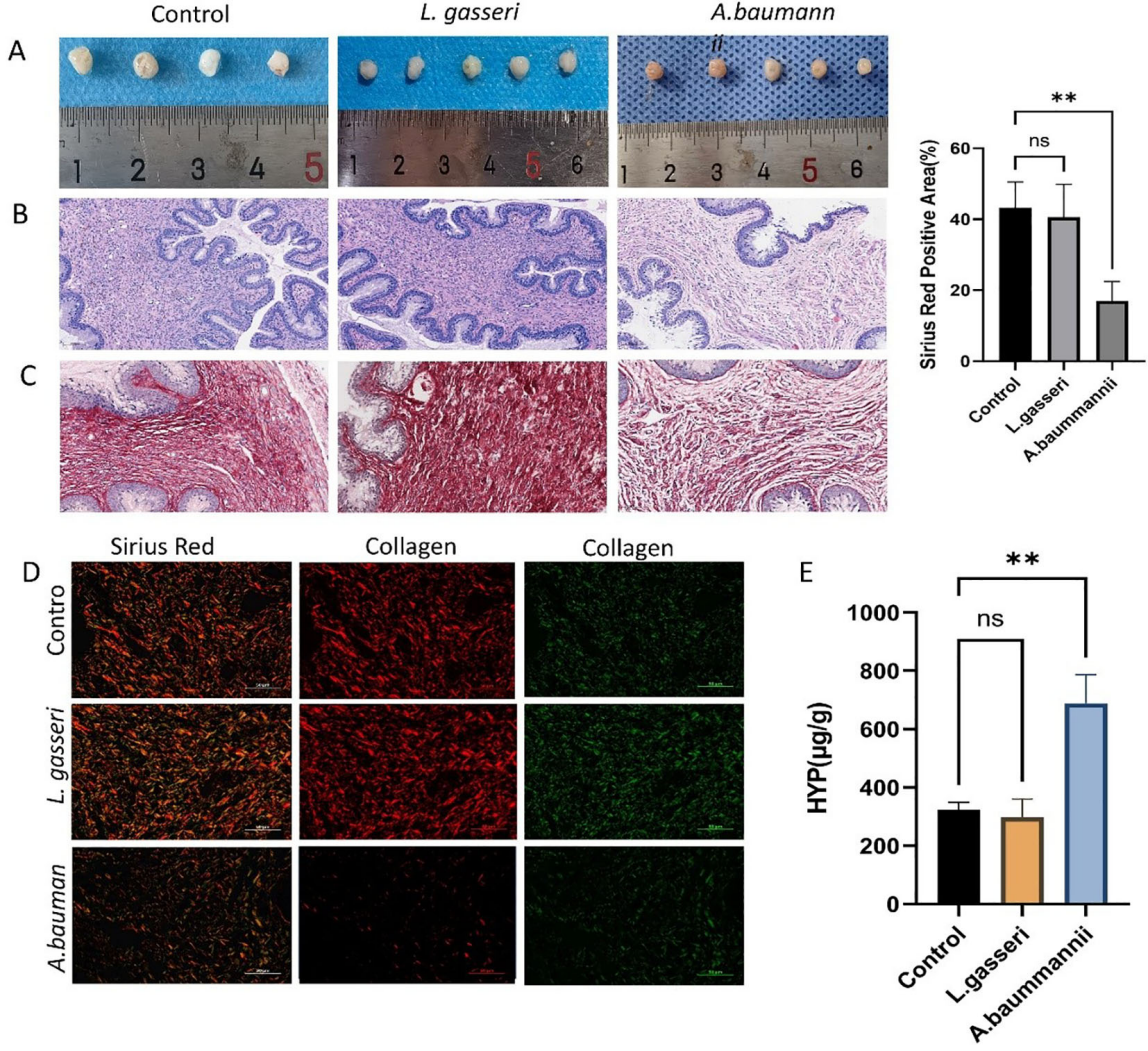
*Acinetobacter* induces cervical collagen degradation in mice, whereas Lactobacillus does not. The cervix of D16 pregnant mice was collected and divided into Acinetobacter group (n=5), Lactobacillus group (n=5) and control group (n=4). There was no difference in the gross structure of the cervix among the three groups **(A)**. H&E **(B)** and Sirius Red staining **(C, D)** revealed sparse, disorganized, and fragmented collagen fibers in the Acinetobacter-inoculated group, whereas the control and Lactobacillus groups showed dense and regularly arranged fibers. The right bar graph shows the quantified percentage of collagen fiber area from three independent experiments. Furthermore, a significant increase in collagen degradation products was observed in the Acinetobacter group **(E)**. Representative images were captured at 10× and 20× magnification. **(B)** Sirius Red staining under light microscopy (red: collagen fibers; blue: nuclei); **(D)** under polarized light microscopy (red: type I collagen; green: type III collagen). Data are mean ± SD. **p < 0.01; ns, not significant.

To investigate whether vaginal colonization by *Acinetobacter* contributes to preterm birth, cervical tissues were collected from mice following intravaginal inoculation with sterile PBS, *Lactobacillus gasseri*, or *Acinetobacter* for comparative analysis. No differences in the gross cervical structure were observed among the three groups (control, n = 4; *Lactobacillus gasseri*, n = 5; *Acinetobacter*, n = 5) (**Figure 3A**).

Hematoxylin and eosin (H&E) staining and Sirius red staining showed that cervical collagen fibers in mice inoculated with *Acinetobacter* were loosely arranged and disorganized. In comparison, collagen fibers in mice inoculated with *Lactobacillus gasseri* or sterile PBS displayed a dense, interwoven network structure (**Figures 3B, C**). Under polarized light microscopy, a significant reduction in cervical collagen fibers was observed in mice inoculated with *Acinetobacter*, with a particularly pronounced decrease in type I collagen fibers (**Figure 3D**).

The cervical level of hydroxyproline (HYP), a degradation product of collagen, was significantly higher in the *Acinetobacter* group than in the control group (*p* < 0.05). In comparison, no significant difference in HYP content was detected between the Lactobacillus gasseri group and the control group (*p* > 0.05) (**Figure 3E**).

### MMP-1 is a differentially expressed gene between premature and normal pregnant women

**Figure 4:**
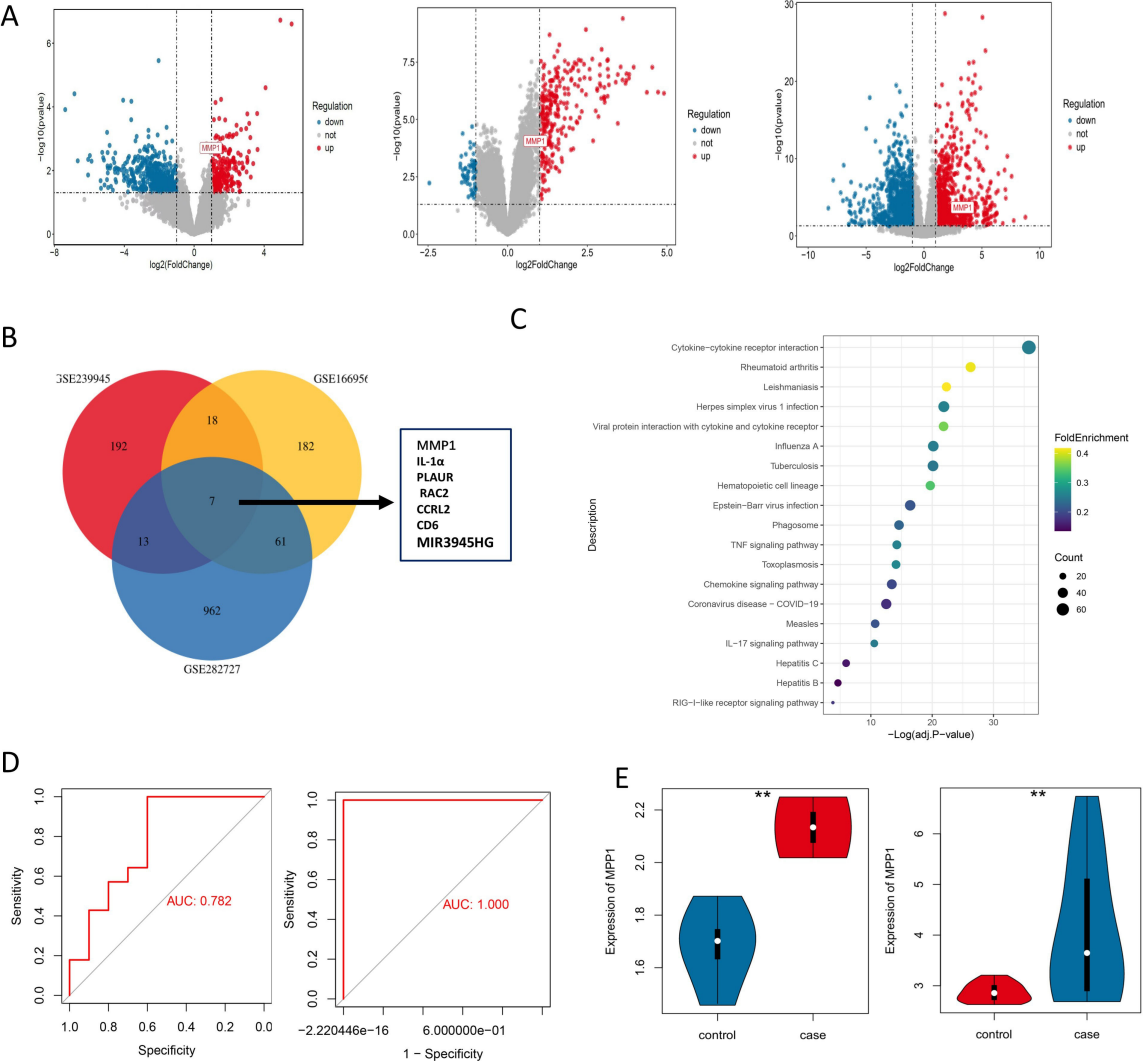
MMP-1 is a differentially expressed gene associated with preterm birth. Differential expression analysis of the datasets *GSE239945*, *GSE166956*, and *GSE282727* showed that MMP-1 was consistently upregulated in preterm birth samples **(A)**. Intersection analysis of the upregulated genes from the three datasets identified seven genes associated with preterm birth, which were enriched in 19 KEGG pathways **(B, C)**. In the *GSE239945* and *GSE166956* datasets, receiver operating characteristic (ROC) curve analysis showed that the area under the curve (AUC) for MMP-1 was 0.782 and 1.000, respectively **(D)**. Differential expression analysis further confirmed that MMP-1 expression differed significantly between the groups **(E)**. **p < 0.01.

To identify genes associated with preterm birth, related datasets (GSE239945, GSE166956, and GSE282727) were obtained from the GEO database (https://www.ncbi.nlm.nih.gov/geo/) for differential expression analysis. All datasets included cervical samples from pregnant women, with normal (term delivery) and preterm birth groups. GSE239945 and GSE166956 were used as the discovery cohort, while GSE282727 served as the validation cohort. The significance threshold was defined as |log^2^(Fold Change)| > 1 and *p*-value < 0.05. The analysis identified 230 upregulated and 532 downregulated genes in GSE239945, 268 upregulated and 59 downregulated genes in GSE166956, and 1,043 upregulated and 1,034 downregulated genes in GSE282727 (**Figure 4A**).

Intersection analysis of the upregulated genes from the three datasets revealed seven commonly upregulated genes associated with preterm birth: *MMP1*, *IL-1α*, *PLAUR*, *RAC2*, *CCRL2*, *CD6*, and *MIR3945HG* (**Figure 4B**). After integrating and removing duplicate genes from the upregulated gene sets, functional enrichment analysis identified 19 significantly enriched KEGG pathways (**Figure 4C**).

Among these genes, matrix metalloproteinase-1 (MMP-1), which is closely associated with cervical remodeling, was selected for further investigation. In the *GSE239945* and *GSE166956* datasets, the area under the ROC curve (AUC) for MMP-1 was 0.782 and 1.000, respectively (**Figure 4D**). Differential expression analysis further confirmed that MMP-1 expression was significantly altered in both datasets (*p* < 0.05; **Figure 4E**).

### *Acinetobacter* induces high expression of MMP-1 in both *in vivo* and *in vitro* models

**Figure 5:**
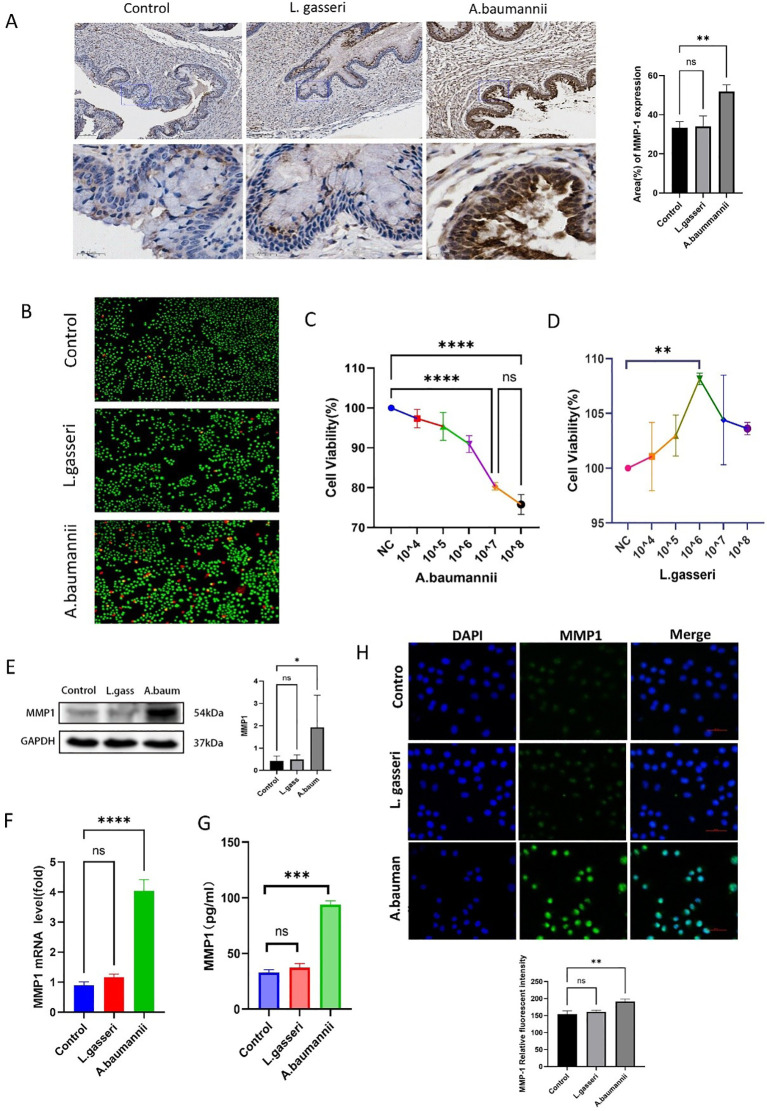
*Acinetobacter* induces elevated MMP-1 expression in both *in vivo* and *in vitro* models. Immunohistochemical staining showed that cervical MMP-1 expression was significantly increased in mice intravaginally inoculated with *Acinetobacter* (n=5) compared with the control group(n=4). In comparison, no significant change was observed in mice inoculated with *Lactobacillus* (n=5) **(A)**, the bar graph on the right shows the quantitative results of the percentage of MMP-1 positive area from three independent experiments. SYTO/PI staining demonstrated a significant increase in the number of dead Hcer Epic cells after co-culture with *Acinetobacter* **(B)**. The optimal colony-forming units (CFU) for co-culture were determined to be 10^7^ CFU for Acinetobacter and 10^6^ CFU for *Lactobacillus* **(C, D)**. Western blot **(E)**, qPCR **(F)**, ELISA **(G)**, and immunofluorescence staining **(H)** consistently showed that *Acinetobacter*, but not *Lactobacillus*, significantly promotes MMP-1 expression in Hcer Epic cells. Representative images were captured at 10× and 40× magnification. In SYTO/PI staining, live cells appear green and dead cells appear red. All data are expressed as mean ± SD (n = 3). *p < 0.05, **p < 0.01, ***p < 0.001, ***p < 0.0001, ns indicates no significant difference.

Immunohistochemical staining showed that MMP-1 expression in the cervical tissue of mice intravaginally inoculated with *Lactobacillus gasseri* did not differ significantly from that of the control group(*p*>0.05). In comparison, mice inoculated with *Acinetobacter* demonstrated significantly increased MMP-1 expression in cervical tissues(*p*<0.05) (**Figure 5A**).

SYTO/PI staining demonstrated a significant increase in the number of dead Hcer Epic cells after co-culture with *Acinetobacter* (**Figure 5B**). According to the CCK-8 assay, the optimal colony-forming units (CFU) for co-culture were determined to be 10^7^ CFU of *Acinetobacter* and 10^6^ CFU of *Lactobacillus gasseri* per 5 × 10^4^ Hcer Epic cells (**Figures 5C, D**). Western blot and qPCR analyses further indicated that, compared with the control group, both MMP-1 protein and mRNA levels were significantly elevated in Hcer Epic cells exposed to *Acinetobacter* (*p* < 0.05). In comparison, exposure to *Lactobacillus gasseri* did not result in a significant change in MMP-1 expression (*p* > 0.05) (**Figures 5E, F**).

ELISA results showed that *Acinetobacter* significantly increased MMP-1 levels in the supernatant of Hcer Epic cells compared with the control group (*p* < 0.05), whereas *Lactobacillus gasseri* had no significant effect (*p* > 0.05) (**Figure 5G**). Immunofluorescence staining further confirmed that *Acinetobacter* promotes MMP-1 expression in Hcer Epic cells (**Figure 5H**).

### *Acinetobacter* facilitates MMP-1 transcription via Snai2 activation

**Figure 6:**
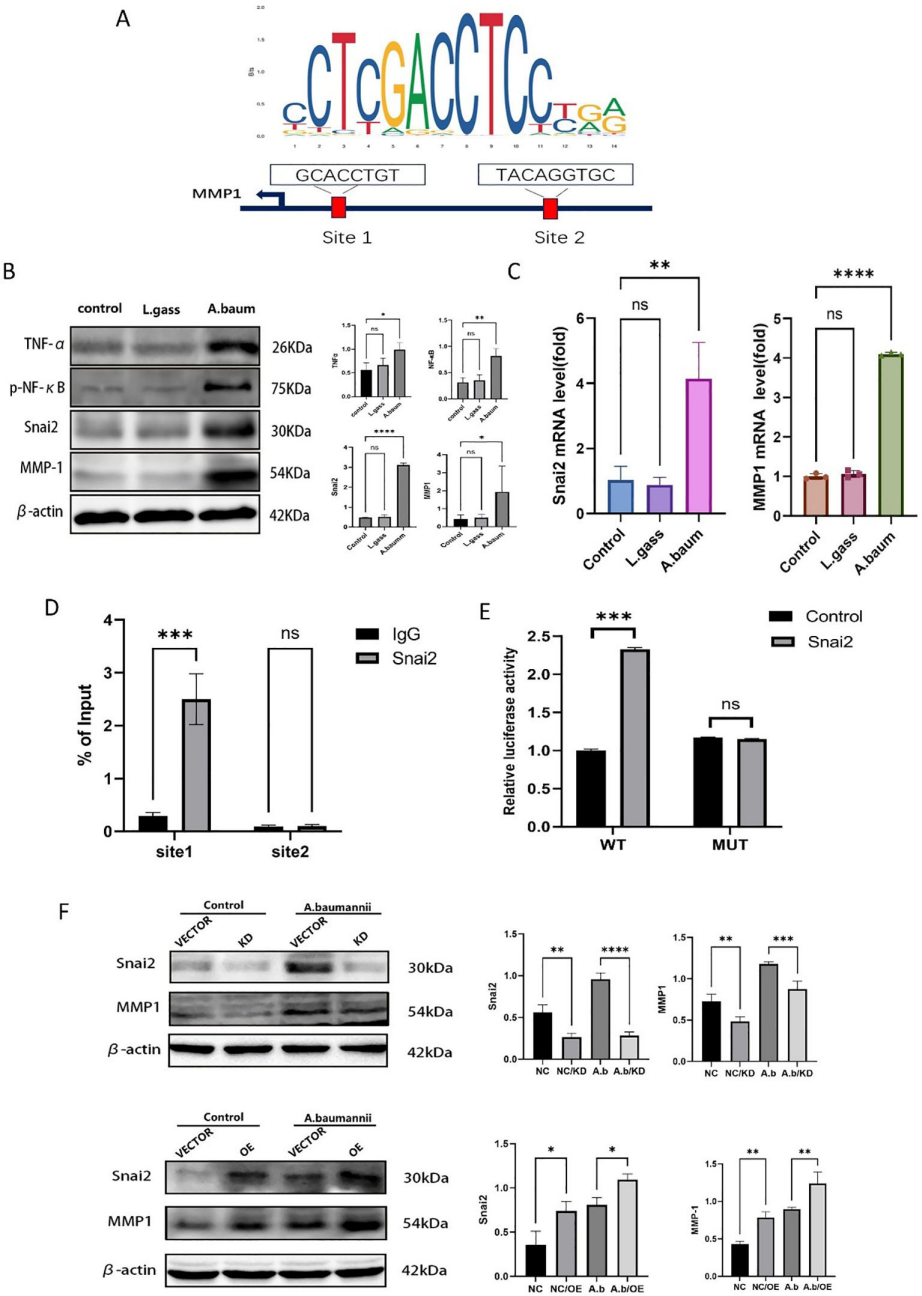
*Acinetobacter* facilitates MMP-1 transcription via Snai2 activation. Bioinformatic analysis predicted potential Snai2 binding motifs within the MMP-1 promoter region **(A)**. Western blot and qPCR analyses showed that co-culture of Hcer Epic cells with *Acinetobacter* significantly increased the expression of both Snai2 and MMP-1 at the protein and mRNA levels **(B, C)**. Chromatin immunoprecipitation (ChIP) assays confirmed that Snai2 binds to site 1 within the MMP-1 promoter **(D)**. Luciferase reporter assays demonstrated that Snai2 specifically regulates the wild-type MMP-1 promoter, whereas no significant effect was observed with the mutant promoter **(E)**. Western blot analysis showed that Snai2 knockdown reduced MMP-1 protein levels, whereas Snai2 overexpression increased MMP-1 levels **(F)**. All data are expressed as mean ± SD (n = 3), *p < 0.05, **p < 0.01, ***p < 0.001, ***p < 0.0001, ns indicates no significant difference.

To identify transcription factors potentially regulating MMP-1 gene expression, the genomic sequence spanning from -2000 bp to +200 bp relative to the transcription start site was retrieved. Putative transcription factor binding sites were predicted using the JASPAR database (https://jaspar.elixir.no/). The search was performed against the JASPAR CORE non−redundant collection of position frequency matrices, applying a relative profile score threshold of 80%. Only predictions with a p−value < 0.05 were considered significant and retained for further analysis. This analysis revealed that Snai2 contains a potential binding motif (GCACCTGT) within the MMP-1 promoter region. (**Figure 6A**). In Hcer Epic cells treated with *Acinetobacter*, both Western blot and qPCR analyses showed significant upregulation of Snai2 and MMP-1 at the protein and mRNA levels (*p* < 0.05) (**Figures 6B, C**).

The ChIP assays further demonstrated that Snai2 binds to the site 1 region of the MMP-1 promoter (**Figure 6D**). Luciferase reporter assays indicated that Snai2 specifically targets the wild-type MMP-1 promoter sequence. In comparison, no binding activity was observed with the mutant sequence (**Figure 6E**). Western blot analysis showed that Snai2 knockdown reduced MMP-1 expression, while overexpression of Snai2 led to increased MMP-1 levels (**Figure 6F**).

These results indicate that Snai2 acts as a transcriptional regulator that promotes MMP-1 expression.

## Discussion

The vaginal microbiota maintains a dynamic equilibrium in which *Lactobacillus* species typically predominate, while other microorganisms remain present at low abundance as opportunistic pathogens. The classical concept of vaginal health emphasizes that *Lactobacillus* species produce lactic acid, maintaining an acidic microenvironment that protects the vagina from invasion by external pathogens (Smith and Ravel, 2017; Chee et al., 2020; Swidsinski et al., 2023). However, advances in next-generation sequencing and metagenomic analyses have revealed substantial heterogeneity in vaginal microbial composition across populations. This variability is influenced by race, ethnicity, geographic location, and significant interindividual differences (Ursell et al., 2012; Saraf et al., 2021). During pregnancy, the vaginal microbiota undergoes characteristic remodeling, generally characterized by reduced microbial diversity and enhanced community stability, which supports a healthy gestational environment (Romero et al., 2014; Gupta et al., 2020). However, dysbiosis of the vaginal microbiota during pregnancy, characterized by decreased *Lactobacillus* abundance and increased colonization by potentially pathogenic bacteria, has been associated with a higher risk of pregnancy complications and adverse birth outcomes (Donders et al., 2011; Petricevic et al., 2014; Zhao et al., 2021; Silvano et al., 2023; Schuster et al., 2024). In this study, 16S rRNA sequencing indicated that *Lactobacillus gasseri* was the dominant species in the vaginal microbiota of the study population, reflecting the known heterogeneity of vaginal microbial communities. However, the relatively small sample size limits the extent to which these findings can be generalized to populations in other geographic regions. Consistent with previous reports, pregnant women who experienced preterm birth demonstrated lower relative abundance of *Lactobacillus* and higher alpha diversity, indicating greater microbial richness and diversity, along with reduced microbiota stability.

Lef Se analysis identified *Acinetobacter* as a characteristic microbial biomarker in the vaginal microbiota of women in the preterm birth group. *Acinetobacter* is a Gram-negative, aerobic, non-fermentative, oxidase-negative *coccobacillus* widely distributed in the natural environment. It can also colonize multiple body sites, including the skin, respiratory tract, gastrointestinal tract, and vagina, where it may function as an opportunistic pathogen (Doughari et al., 2011; Wong et al., 2017). Studies specifically examining *Acinetobacter* within the vaginal microbiota remain limited. Previous reports suggest that this genus may even represent a dominant bacterial group in the upper reproductive tract environment (Winters et al., 2019; Kaakoush et al., 2022; Canha-Gouveia et al., 2023).

Regarding its pathogenic potential, *Acinetobacter* has been isolated in cases of aerobic vaginitis during pregnancy (Nguyen et al., 2022), has been associated with embryo implantation failure (Zhang et al., 2024), and has been linked to miscarriage and preterm birth (Aivazova et al., 2010; McGrath et al., 2011; He et al., 2013). However, these observations are largely based on case reports and have not been validated in large-scale studies.

This study investigated the potential pathogenic role of *Acinetobacter* using both *in vivo* and *in vitro* models, with *Lactobacillus gasseri* serving as a microbial control. Our findings demonstrated, at both the animal and cellular levels, that *Acinetobacter* promotes MMP-1 expression, leading to degradation of cervical collagen fibers and weakening of cervical mechanical support, representing a potential risk factor for preterm birth. In comparison, *Lactobacillus gasseri* showed no pathogenic effects. The pathogenic activity of *Acinetobacter* may be linked to its LPS, which can interact with Toll-like receptors on cervical epithelial cells, initiating inflammatory signaling cascades. It should be noted that *Acinetobacter* acts as a complete microorganism within the host, and our study did not distinguish between the biological effects of the bacterium itself and those of its secreted metabolites. The ability of microorganisms in the lower genital tract to trigger preterm birth depends on multiple interacting factors, including host immune responses, microbial virulence and load, microbial community interactions, and host–microbe interactions. Therefore, animal experiments focusing on a single microorganism cannot definitively determine its capacity to induce preterm birth. Although our findings cannot conclusively establish vaginal *Acinetobacter* as a direct cause of preterm birth, they demonstrate that this bacterium is capable of inducing cervical collagen degradation. It is conceivable that, within a dysbiotic vaginal ecosystem, the cumulative effects of multiple microorganisms with similar pathogenic properties may substantially increase the risk of preterm birth. Another limitation of this study is that quadrupedal animal models do not fully replicate the gravitational and biomechanical forces experienced by the human cervix during upright posture and locomotion.

Our results indicate that *Acinetobacter* induces MMP-1 expression in both mice and Hcer Epic cells. MMP-1 cleaves the triple-helical region of collagen fibers at the Gly775–Leu/Ile776 site and functions as an initiator of collagen degradation (Gross and Lapiere, 1962; Brinckerhoff and Matrisiam, 2002). This enzyme participates in numerous physiological and pathological processes, including scar repair, tissue remodeling, and tumor metastasis. In this study, mice with elevated MMP-1 expression showed loosened and disorganized cervical collagen fibers, significantly increased collagen degradation products, and more pronounced degradation of type I collagen fibers, further highlighting MMP-1’s strong collagen-degrading capacity. The expression of MMP-1 is tightly regulated at both transcriptional and post-translational levels. At the transcriptional level, MMP-1 expression can be induced by various cytokines and growth factors, many of which regulate gene transcription through the AP-1 binding site within the MMP-1 promoter (Sternlicht and Werb, 2001). The Ets and AP-1 binding sites in the MMP-1 promoter are separated by only 9 nucleotides, a spatial arrangement that facilitates coordinated regulation by additional transcription factors (Gutman and Wasylyk, 1990; Pendás et al., 1997). After translation, MMP-1 is synthesized as an inactive zymogen that requires activation. This activation may occur through modification of the cysteine residue in its prodomain by reactive oxygen species or through proteolytic cleavage, after which the enzyme is secreted extracellularly to exert its biological activity. In our experiments, stimulation of Hcer Epic cells with Acinetobacter led to TNF-α production. TNF-α not only enhanced MMP-1 transcription but also impaired mitochondrial function, increasing the generation of reactive oxygen species (ROS). These ROS subsequently oxidized the cysteine residue in the MMP-1 prodomain, promoting further activation of the enzyme.

This study identified Snai2 as a transcription factor involved in regulating MMP-1 expression in human cervical epithelial cells. Snai2, a member of the SNAIL family of zinc-finger transcription factors, is widely recognized as a key regulator of epithelial–mesenchymal transition (EMT). A classic mechanism involves Snai2-mediated repression of E-cadherin and other adhesion molecules, a process that enhances cellular migratory and invasive capabilities (Batlle et al., 2000; Nieto, 2002; Thiery and Sleeman, 2006). With continued research, the functional spectrum of Snai2 has expanded considerably (Zhou et al., 2019). In tumor biology, Snai2 and MMP-1 often act synergistically: Snai2 enhances the ability of tumor cells to degrade extracellular matrix components, facilitating metastasis (Mahmoudian et al., 2021). Previous studies have demonstrated that Snai2 is regulated by the NF-κB signaling pathway (Wu et al., 2009). In the present study, experimental results indicated that co-culture of *Acinetobacter* with HcerEpic cells activates the TNF-α/NF-κB signaling pathway. Therefore, it can be deduced that *Acinetobacter* activates Snai2 in our model. (**Figure 6B**). Moreover, the ChIP and dual-luciferase reporter assays further confirmed the presence of a Snai2 binding motif (GCACCTGT) within the MMP-1 promoter. Moreover, knockdown and overexpression experiments showed that modulation of Snai2 correspondingly altered MMP-1 expression. Collectively with the established transcriptional regulatory mechanisms of MMP-1, these findings indicate that Snai2 directly participates in MMP-1 transcriptional regulation in Hcer Epic cells. Although our data show that Snai2 influences MMP-1 expression, the AP-1 binding site remains the principal regulatory element in the MMP-1 promoter, with Snai2 acting as a cooperative transcriptional regulator.

It is important to note that this study utilized single representative strains of each species: *Acinetobacter* sp. (BNCC 337486) and *Lactobacillus gasseri* (BNCC 135322). Therefore, the conclusions drawn from this work are strictly applicable to these specific strains and cannot be automatically generalized to all members of the genus *Acinetobacter* or *Lactobacillus*, nor to all Gram-negative bacteria. Future comparative studies using diverse clinical and laboratory isolates are required to establish the genus-level validity of these findings.

## Conclusion

This study identified distinct differences in the vaginal microbiota between women with preterm birth and those with normal pregnancies. The findings indicate that vaginal colonization by *Acinetobacter* can induce elevated expression of MMP-1, resulting in degradation of cervical collagen fibers and potentially contributing to the development of preterm birth. *Acinetobacter* activates Snai2, which in turn stimulates MMP-1 transcription.

This study provides novel evidence that Snai2 regulates MMP-1 expression in human cervical epithelial cells. These results suggest that targeting the cooperative regulatory role of Snai2 may represent a potential therapeutic approach for preventing or treating threatened preterm birth associated with cervical collagen degradation.

## Data Availability

The data presented in the study are deposited in the Figshare repository. The DOI for this dataset is https://doi.org/10.6084/m9.figshare.32542602.
